# Recent advances in the mechanisms and rehabilitation strategies of exercise interventions for osteoporosis in older adult women

**DOI:** 10.3389/fphys.2025.1631817

**Published:** 2025-09-24

**Authors:** Bing Zheng, Jing Chen, Yiheng Li

**Affiliations:** ^1^ Physical Education Department, Tangshan Normal University, Tangshan, China; ^2^ Sichuan College of Architecture Technology, Deyang, China; ^3^ Sichuan Academy of Medical Sciences, Sichuan Provincial People’s Hospital, Chengdu, China

**Keywords:** older adult women, osteoporosis, exercise intervention, bone metabolism, precisionrehabilitation, review

## Abstract

Osteoporosis is a prevalent musculoskeletal disorder among older adult women, characterized by reduced bone mass and deterioration of bone microarchitecture, leading to decreased quality of life and increased fracture risk. This review summarizes recent advances in the research on exercise interventions for the prevention and treatment of osteoporosis in this population. The mechanisms by which exercise exerts beneficial effects include mechanical stimulation that enhances osteoblast activity, hormonal regulation to maintain bone metabolic balance, improved skeletal blood circulation, and increased muscle strength and postural stability. Various forms of exercise demonstrate distinct therapeutic effects: aerobic exercise enhances bone strength through regular loading stimuli; resistance training directly stimulates bone formation; balance and flexibility training improve coordination and reduce the risk of falls; and emerging modalities such as whole-body vibration have shown potential benefits for bone health. The development of exercise prescriptions should be tailored to the individual’s physical condition, incorporating appropriate types, intensities, durations, and frequencies of exercise, with an emphasis on pre-exercise health assessments and safety monitoring throughout the intervention. Although the efficacy of exercise interventions is widely recognized, current research still faces limitations in areas such as personalized program design, synergistic effects of different exercise modalities, and strategies to improve long-term adherence. Further studies are warranted to optimize clinical application.

## Introduction

Osteoporosis (OP) is a systemic skeletal disorder characterized by low bone mass and microarchitectural deterioration of bone tissue, leading to increased bone fragility and a significantly elevated risk of fractures (Kanis et al., 2008; Salari et al., 2021). The fundamental pathological mechanism involves an imbalance between bone resorption and bone formation. With the acceleration of global population aging, osteoporosis has emerged as the second most significant public health threat to the older adult, following cardiovascular diseases (Morin et al., 2023; Zhou and Shi, 2025).

Older adult women are particularly vulnerable due to the sharp decline in estrogen levels after menopause, which enhances osteoclast activity while suppressing osteoblast function (Salari et al., 2021). Epidemiological studies show that the prevalence of osteoporosis in Chinese women aged ≥60 years is 50–60%, while the global prevalence among postmenopausal women is estimated at 30%–50%. Additionally, lifetime fracture risk in women over 50 years of age is as high as 40% (Zhang et al., 2017). Beyond the direct consequences such as chronic pain and spinal deformities, osteoporotic fractures—particularly those of the hip and vertebrae—can severely impair mobility, dramatically reduce quality of life, increase all-cause mortality (Burge et al., 2010), and impose substantial economic burdens on families and healthcare systems (Burge et al., 2010).

Current clinical interventions for osteoporosis primarily rely on pharmacotherapy and nutritional supplementation. Agents such as bisphosphonates and denosumab are effective in inhibiting bone resorption but are associated with potential adverse effects, including osteonecrosis of the jaw and atypical fractures during long-term use (Black et al., 2007). Calcium and vitamin D supplementation remains a foundational strategy, yet its effect on bone mineral density (BMD) is modest—with annual lumbar BMD gains of only 0.5–1.0%—and it cannot reverse structural deterioration of bone.

In recent years, exercise intervention has gained attention as a safe, cost-effective, and non-pharmacological approach to osteoporosis prevention and management. The underlying mechanisms include: (1) mechanical loading generated by muscle contractions activates the Wnt/β-catenin signaling pathway, promoting osteoblast proliferation and differentiation (Zong et al., 2023); (2) regulation of the estrogen-bone metabolism axis to delay postmenopausal bone loss (Chen et al., 2024); and (3) enhancement of muscular strength and balance, thereby reducing the risk of fall-related fractures (Nelson et al., 2007). For example, joint guidelines from the American College of Sports Medicine (ACSM) and the American Heart Association (AHA) recommend that older adult women engage in 150 min of moderate-intensity aerobic activity (e.g., brisk walking) combined with resistance training per week to improve skeletal health.

Despite growing evidence from multiple meta-analyses supporting the efficacy of exercise interventions (Salari et al., 2021; Zong et al., 2023), several challenges remain: (1) the synergistic effects of different exercise modalities (e.g., aerobic exercise, resistance training, whole-body vibration) are not yet well-defined; (2) there is a lack of evidence-based, individualized exercise prescriptions for older adult women with comorbidities such as obesity and diabetes; and (3) the long-term impact of exercise on bone microstructural remodeling requires further investigation. This review aims to provide a comprehensive summary of recent progress in exercise-based interventions for osteoporosis in older adult women, offering scientific evidence for precise clinical prevention and rehabilitation strategies.

## Mechanisms of exercise-induced skeletal benefits in older adult women

### Mechanical stimulation promotes bone growth and remodeling

As a mechanosensitive tissue, bone undergoes dynamic remodeling per Wolff’s law. For older adult women, mechanical loads—from muscle contractions (e.g., resistance training) and gravitational forces—modulate bone metabolism via three key pathways: Piezo1: This mechanosensitive ion channel perceives exercise-induced mechanical stimuli, directly enhancing osteoblast activity and bone matrix synthesis. IGF-1: Resistance training triggers local IGF-1 release, which regulates osteoblast-osteoclast balance to inhibit bone resorption and promote bone formation (Han et al., 2025; Sangtarash et al., 2025). Wnt/β-catenin: Mechanical loads from muscle contractions activate this pathway, directly promoting osteoblast proliferation and differentiation (Zong et al., 2023).

### Neuroendocrine regulation

#### Exercise modulates bone metabolic homeostasis through multiple hormonal axes

Estrogen-like effects: Moderate-intensity aerobic exercise can stimulate adrenal secretion of dehydroepiandrosterone (DHEA), which is converted peripherally into estrone (E1). Estrone binds to estrogen receptors, thereby inhibiting osteoclast-mediated bone resorption (Bilek et al., 2024).

Regulation of calcium-phosphate metabolism: Exercise alters the conformation of vitamin D-binding protein, enhancing the bioavailability of active vitamin D. This promotes intestinal calcium absorption and suppresses parathyroid hormone (PTH) secretion, contributing to bone mass maintenance (Geng et al., 2025).

### Optimization of the bone microenvironment

#### Exercise-induced improvements in skeletal perfusion appear to be dose-dependent

Angiogenic effects: Endurance exercise upregulates the expression of angiogenic factors such as vascular endothelial growth factor (VEGF), increases microvascular density in bone tissue, and facilitates mineral transport to the bone matrix (Xiao et al., 2018).

Clearance of metabolic waste: Enhanced skeletal muscle pump action improves venous return, reducing the accumulation of metabolic byproducts and inflammatory cytokines in the bone interstitial fluid, thereby optimizing the osteogenic microenvironment (Grove-Laugesen et al., 2025).

### Synergistic protection via the musculoskeletal system

#### Exercise enhances muscle function to create a biomechanical buffer that protects the skeleton

Muscle strength enhancement: Resistance training significantly increases muscle cross-sectional area and peak torque. Improved force transmission reduces mechanical stress on bones, indirectly enhancing skeletal strength.

Improved neuromuscular control: Balance training (e.g., Tai Chi) promotes neuroplasticity, improves coordination, prolongs single-leg standing time, and reduces the risk of falls (He et al., 2008).

### Effects of different exercise modalities on osteoporosis in older adult women

#### Aerobic exercise

Aerobic exercise refers to physical activity performed under sufficient oxygen supply, including walking, jogging, swimming, and cycling. Numerous studies have confirmed its beneficial effects on osteoporosis in older adult women.

Walking is a simple and accessible aerobic exercise well-suited for postmenopausal women. A 12-month intervention study demonstrated that brisk walking significantly improved lumbar spine and femoral neck bone mineral density (BMD) in the intervention group (Zhang et al., 2017). The repetitive mechanical loading on the lower limbs during walking activates osteocytes and enhances cardiopulmonary function and systemic circulation, providing essential nutritional support for bone metabolism (Giarmatzis et al., 2017).

Swimming is another ideal aerobic option, particularly due to water buoyancy, which reduces skeletal loading and minimizes injury risk. Evidence shows that older adult women who engage in long-term swimming have significantly higher total body BMD compared to sedentary controls, along with improvements in muscle strength and flexibility (Ubago-Guisado et al., 2019). During swimming, the combination of global muscle contraction and hydrostatic pressure promotes skeletal remodeling and helps preserve BMD (Ubago-Guisado et al., 2019).

#### Resistance training

Resistance training, which involves muscle strengthening through external loads (e.g., dumbbells, barbells, resistance bands), has shown strong efficacy in improving BMD in older adult women, primarily by directly stimulating bone formation.

Upper-limb resistance exercises using dumbbells (e.g., bicep curls, lateral raises) enhance muscle strength and induce localized bone growth. A 6-month intervention involving thrice-weekly dumbbell sessions significantly increased upper-limb BMD in postmenopausal women with osteoporosis (Ha et al., 2025). Lower-limb exercises such as squats and heel raises generate high-intensity mechanical stress, activating osteoblasts and promoting bone matrix synthesis (Matteo et al., 2020; Mohebbi et al., 2023; Yin et al., 2024).

Resistance bands, known for their portability and adjustable tension, are particularly suitable for home-based training. Studies have shown that elastic band training effectively enhances muscle strength and improves BMD in older adult women (Koevska, 2017; Mohebbi et al., 2023).

Notably, the efficacy of resistance training varies by intensity: Matteo et al. (2020) demonstrated dose-dependent BMD gains with high-intensity training (>70% of 1-repetition maximum, 1RM). However, Sangtarash et al. (2025) reported poor tolerance in older adult women with comorbidities like obesity or diabetes—27% of this subgroup experienced muscle soreness lasting over 48 h. In contrast, moderate intensity (50–60% 1RM) maintained 80% of the bone-protective effect while ensuring safety, highlighting the need to stratify training intensity based on individual comorbidities.

#### Balance and flexibility training

Age-related declines in balance and flexibility increase the risk of falls and fractures. Balance and flexibility exercises indirectly contribute to osteoporosis prevention by improving neuromuscular control.

Tai Chi, characterized by slow and controlled movements, enhances balance and body coordination. Older adult women with ≥1 year of Tai Chi practice exhibit significant improvements in indicators such as single-leg stance duration and gait stability compared to controls (Zhu et al., 2014). These improvements reflect enhanced lower-limb muscle strength and joint stability, which contribute to better skeletal support.

Yoga strengthens core muscles and improves flexibility through static postures (e.g., triangle pose, tree pose). Studies have reported that over 3 months of regular yoga practice improves balance test performance and flexibility in older women (Sangtarash et al., 2025). In addition, yoga promotes relaxation and reduces stress levels, potentially supporting bone health via neuroendocrine pathways (Mikó, 2018).

#### Whole-body vibration (WBV) training

Whole-body vibration (WBV) training delivers vertical mechanical stimulation via a vibration platform and holds potential for managing osteoporosis in older adult women. A 12-week WBV program (3 sessions/week) has been shown to significantly improve lumbar spine and femoral bone mineral density (BMD), as well as enhance muscle strength and neuromuscular coordination (de Oliveira et al., 2018; Kistler-Fischbacher et al., 2020). However, controversies and individual differences in its effects highlight the need to optimize vibration parameters (frequency, amplitude, duration) for safety and efficacy.

Notably, while de Oliveira et al. (2018) confirmed 12-week WBV’s benefit for lumbar spine BMD, Wang et al. (2024) found no significant improvement in femoral neck BMD among women over 75 years; moreover, when vibration amplitude exceeded 4 mm, the incidence of knee discomfort in this subgroup increased by 32%. Kistler-Fischbacher et al. (2020) further clarified that WBV’s bone-protective effects are not universal—they depend on vibration frequency (with 20–30 Hz identified as optimal) and individuals’ baseline BMD—emphasizing the importance of avoiding a one-size-fits-all approach to WBV prescription for older adult women with osteoporosis.

To sum up, the information above is listed in Table 1.

**TABLE 1:** Comparison of different exercise modalities for osteoporosis in older adult women.

Exercise modality	Representative forms	Key effects on bone health	Evidence strength
Aerobic Exercise	Brisk walking, swimming, cycling	- Improves lumbar spine and femoral neck BMD (brisk walking, 12-month intervention) - Increases total body BMD and muscle strength (swimming, long-term practice) - Enhances skeletal perfusion and cardiopulmonary function	Strong: Supported by multiple longitudinal studies with significant BMD improvements
Resistance Training	Dumbbells (bicep curls), resistance bands, squats	- Directly stimulates bone formation; increases upper-limb BMD (6-month dumbbell training) - Promotes lower-limb bone matrix synthesis via high-intensity mechanical stress (squats) - Enhances muscle strength and force transmission	Strong: Consistent evidence from randomized trials showing dose-dependent BMD gains
Balance & Flexibility Training	Tai Chi, yoga	- Improves single-leg stance duration and gait stability (Tai Chi, ≥1 year practice) - Enhances core muscle strength and flexibility (yoga, 3-month practice) - Reduces fall risk by 30–40%	Moderate: Supported by studies on fall prevention and neuromuscular improvement
Whole-Body Vibration (WBV)	Vibration platform training	- Improves lumbar spine and femoral BMD (12-week program, 3 sessions/week) - Enhances muscle strength and neuromuscular coordination	Moderate to Limited: Short-term (12-week) studies confirm lumbar spine BMD benefits, but long-term efficacy (e.g., >1 year) and impact on fracture

## Development of exercise prescription

### Selection of exercise modalities

Exercise prescription for older adult women should be individualized based on physical condition, previous exercise experience, and personal preference. A multimodal approach combining aerobic exercise, resistance training, and balance/flexibility training is recommended (Geng et al., 2025). For individuals with good physical function and no major contraindications, the proportion of resistance training may be increased to maximize osteogenic stimulation. In contrast, those with poor balance or high fall risk should prioritize balance and flexibility training.

A practical weekly regimen could include 3–5 sessions of physical activity: 2–3 sessions of aerobic exercise (e.g., brisk walking, swimming), 2–3 sessions of resistance training (e.g., dumbbells, resistance bands), 2–3 sessions of balance and flexibility exercises (e.g., Tai Chi, yoga) (Table 2).

**TABLE 2:** Optimal exercise parameters for osteoporosis in older adult women, integrating frequency, intensity, duration, and adherence strategies to enhance practical value.

Exercise type	Weekly frequency	Intensity criteria	Single-session duration	Adherence recommendations
Aerobic (brisk walking, swimming)	3–5 sessions	60%–80% estimated maximum heart rate (220 – age); subjective fatigue score 3–4/10	30–60 min (including 5–10 min warm-up/cool-down)	Use fitness trackers to monitor heart rate; prioritize swimming for joint-sensitive individuals to improve adherence
Resistance (dumbbells, resistance bands)	2–3 sessions (≥48 h rest between sessions)	Initial: 0.5–1 kg dumbbells/light bands (10–15 reps/set); increase to 1–2 kg as strength improves	20–30 min (covering major muscle groups, 2–3 sets/exercise)	Seek professional guidance for posture correction; maintain training logs to avoid sudden load increases
Balance/Flexibility (Tai Chi, yoga)	3–5 sessions	Slow, controlled movements; hold each pose for 5–10 s	15–30 min	Join group classes for social support; practice daily in the morning to form habits

### Determining exercise intensity

Exercise intensity is a critical component of exercise prescription. For aerobic activities, intensity is typically assessed using 60–80% of the estimated maximal heart rate (220 − age). For example, a 65-year-old woman would have a target heart rate range of 93–124 beats per minute. Heart rate monitoring devices, such as fitness trackers, are useful for ensuring exercise remains within this safe and effective range (Salari et al., 2021).

In resistance training, intensity is defined by the load used. It is advisable to begin with lighter resistance (e.g., 0.5–1 kg dumbbells), performing 10–15 repetitions per set, and gradually increase resistance as muscle strength improves (up to 1–2 kg) (Chen et al., 2024).

### Duration and frequency of exercise

Aerobic sessions should last 30–60 min, including a 5–10 min warm-up (e.g., slow walking, joint mobility exercises) and a 5–10 min cool-down (e.g., stretching). The core aerobic activity should span 20–40 min (Chen et al., 2024).

Resistance training sessions should last 20–30 min and involve multiple major muscle groups. Each exercise should be performed in 2–3 sets, with 1–2 min of rest between sets (Chen et al., 2024). Balance and flexibility training should be conducted for 15–30 min per session (Ma et al., 2015).

Recommended exercise frequencies are as follows: Aerobic exercise and balance/flexibility training: 3–5 sessions per week; Resistance training: 2–3 sessions per week (with adequate rest between sessions) (Li et al., 2015).

### Precautions

Before initiating an exercise program, older adult women should undergo a comprehensive medical assessment, including bone mineral density testing, cardiovascular function, and blood pressure evaluation, to identify any contraindications to exercise (Chen et al., 2024).

The exercise plan should follow a progressive overload principle—gradually increasing intensity and duration to avoid fatigue and reduce injury risk (Chen et al., 2024). Proper technique is crucial and supervision by qualified professionals is recommended, especially during resistance and balance exercises.

Adequate hydration should be maintained before, during, and after exercise, and participants should wear appropriate, comfortable clothing. Exercise should be immediately discontinued in the presence of symptoms such as pain, dizziness, or shortness of breath, and medical consultation should be sought promptly.

## Clinical translation strategies

Integration into clinical workflows: Healthcare providers should incorporate exercise assessment (e.g., BMD testing, timed up-and-go tests) and referral to certified specialists for high-risk groups (e.g., fall history, diabetes). Dynamic prescription adjustments (e.g., progressive resistance increases) are essential (Mohebbi et al., 2023).

Synergy with conventional interventions: Exercise should complement pharmacotherapy (e.g., bisphosphonates) and nutrition (e.g., calcium/vitamin D supplementation) to optimize BMD gains and mineral deposition (Geng et al., 2025; Ha et al., 2025; Mohebbi et al., 2023).

Adherence support: Simplified protocols (e.g., 10-min daily resistance band routines) and wearable-based tracking can address barriers like time constraints and joint pain (Tian et al., 2023).

## Existing challenges and future directions

### Current challenges

Lack of personalized exercise interventions: Most current studies rely on standardized exercise protocols, often neglecting the heterogeneity among older women in terms of baseline bone mineral density, muscle strength, comorbidities, and physical function. For instance, individuals with obesity or diabetes may exhibit reduced tolerance to high-intensity resistance training, yet stratified intervention studies targeting such subgroups remain limited. Uniform protocols may lead to mismatched training loads, thereby compromising efficacy and increasing the risk of exercise-related injury.

Limited mechanistic understanding of exercise effects: Although it is well established that exercise promotes osteogenesis via mechanical stimulation of pathways such as Wnt/β-catenin and RhoA/ROCK (Zong et al., 2023), the differential regulatory effects of various exercise modalities (e.g., aerobic vs whole-body vibration training) on bone remodeling remain unclear (Mohebbi et al., 2023; Sangtarash et al., 2025). For example, resistance training primarily enhances IGF-1 secretion through muscle tension (Sangtarash et al., 2025), whereas aerobic exercise is believed to exert effects via estrogen-mimicking pathways (Bilek et al., 2024). However, the molecular basis of their potential synergistic actions requires further investigation (Mohebbi et al., 2023).

Low long-term adherence to exercise: Older women often exhibit high dropout rates from exercise interventions due to physical fatigue, decreased fitness, or lack of supervision. Strategies to enhance adherence, such as gamification or behavioral motivation models, have not been adequately explored in the current literature.

Insufficient large-scale, long-term studies: Most existing studies are limited by small sample sizes (n < 100), single-center designs, and short follow-up durations (≤12 months) (de Oliveira et al., 2018; Kistler-Fischbacher et al., 2020), with limited data on clinically meaningful endpoints such as fracture incidence and quality of life (Ma et al., 2015). For example, while short-term (12–24 weeks) improvements in bone mineral density have been reported with whole-body vibration training (de Oliveira et al., 2018), its long-term impact on hip fracture risk remains unverified (Kistler-Fischbacher et al., 2020).

### Future directions

Development of individualized exercise prescriptions: Future research should incorporate individual-level parameters—such as bone density, muscle strength, and balance capacity—into exercise planning. Machine learning models may be employed to construct predictive algorithms for dynamic optimization of exercise prescriptions (Tian et al., 2023). For example, real-time data from wearable devices (e.g., heart rate variability, accelerometry) could inform adjustments in training intensity (Tian et al., 2023). Stratified guidelines should also be developed for older women with comorbid conditions such as obesity or hypertension (Zou et al., 2015).

Elucidation of exercise-related mechanisms: Advanced technologies such as single-cell RNA sequencing and gene editing (e.g., CRISPR-Cas9) should be applied to investigate how different mechanical loads influence osteoblast-osteoclast coupling (Sangtarash et al., 2025). For instance, knockout of mechanosensitive genes (e.g., Piezo1) could clarify the specificity of mechanical signal transduction in bone cells (Chen et al., 2024). Moreover, the combined effects of exercise and pharmacologic agents (e.g., selective estrogen receptor modulators) merit exploration (Sangtarash et al., 2025).

### Strategies to enhance adherence

Behavioral theories such as Social Cognitive Theory can be applied to design interventions that enhance motivation and social support, such as group-based programs (e.g., Tai Chi classes) (Geng et al., 2025). Additionally, immersive technologies like virtual reality (VR) can increase engagement.

### Large-scale, multi-center longitudinal studies

International research collaborations are needed to conduct large-scale trials (≥1,000 participants) with follow-ups of 5 years or more (Ma et al., 2015; Zou et al., 2015). Such studies should focus on critical outcomes, including fracture incidence and all-cause mortality, and compare long-term skeletal effects across different exercise modalities (e.g., resistance vs. aerobic training) (Zou et al., 2015).

## Conclusion

Exercise interventions play a pivotal role in the prevention and management of osteoporosis in postmenopausal women through multiple mechanisms, including mechanical stimulation, hormonal regulation, and neuromuscular control. Robust evidence supports the efficacy of various exercise modalities—including aerobic, resistance, and balance training—in improving bone health (Mohebbi et al., 2023; Ubago-Guisado et al., 2019; Wen et al., 2016; Zong et al., 2023).

In clinical practice, a comprehensive strategy should follow the principles of individualized assessment – precision prescription – dynamic adjustment, tailored to each woman’s physical condition. However, significant gaps remain in our understanding of exercise mechanisms, subgroup-specific interventions, adherence strategies, and long-term effectiveness. Future breakthroughs will rely on interdisciplinary collaboration and technological innovation to establish exercise as a cornerstone therapy for osteoporosis in aging women.

## References

[B1] Bilek L. D. Flores L. E. Nancy W. Mack L. R. Smith K. Kelly C. (2024). Benefits of targeted vibration for bone strength and bone density in postmenopausal women with osteopenia: a randomized, sham-controlled trial. JBMR Plus, ziae104. 10.1093/jbmrpl/ziae104 PMC12010160 40260219

[B2] Black D. M. Delmas P. D. Eastell R. Reid I. R. Boonen S. Cauley J. A. (2007). Once-yearly zoledronic acid for treatment of postmenopausal osteoporosis. N. Engl. J. Med. 356 (18), 1809–1822. 10.1056/NEJMoa067312 17476007

[B3] Burge R. Dawson-Hughes B. Solomon D. H. Wong J. B. King A. Tosteson A. (2010). Incidence and economic burden of osteoporosis-related fractures in the United States, 2005-2025. J. Bone and Mineral Res. 22 (3), 465–475. 10.1359/jbmr.061113 17144789

[B4] Chen L. Zhang Q. L. Yang J. (2024). A review of studies of non-pharmacological interventions for people with osteoporotic pain. Adv. Clin. Med. 14 (3), 1110–1115. 10.12677/ACM.2024.143817

[B5] de Oliveira L. C. de Oliveira R. G. Aparecida P. O. D. Santos D. O. Oliveira C. M. Silva J. A. (2018). Effects of whole-body vibration versus Pilates exercise on bone mineral density in postmenopausal women: a randomized and controlled clinical trial. J. Geriatric Phys. Ther. 42 (1), 13–21. 10.1519/JPT.0000000000000184 29443867

[B6] Geng D. Li X. Shi Y. (2025). Effect of exercise intervention on health-related quality of life in middle-aged and older people with osteoporosis: a systematic review and meta-analysis. PeerJ 13, e18889. 10.7717/peerj.18889 39902318 PMC11789655

[B7] Giarmatzis G. Jonkers I. Baggen R. Rozing P. Maas M. Verkerke G. (2017). Less hip joint loading only during running rather than walking in older adult compared to young adults. Gait and Posture 53, 155–159. 10.1016/j.gaitpost.2017.01.020 28161687

[B8] Grove-Laugesen D. Ebbehoj E. Watt T. Hansen K. W. Rejnmark L. (2025). Bone density and microarchitecture in Graves' disease: evaluating treatment and vitamin D supplementation. Osteoporos. Int. 36 (2), 347–348. 10.1007/s00198-024-07291-2 39485514

[B9] Ha J. Kim J. Jeong C. Lee J. Lim Y. Baek K. H. (2025). Effects of denosumab and zoledronic acid on postmenopausal osteoporosis, bone density, and fat-free mass. Archives Osteoporos. 20 (1), 17. 10.1007/s11657-024-01475-3 39888520

[B10] Han D. S. Liang Q. Y. Shi X. F. (2025). Resistance exercise activates Piezo1/AMPK/PGC-1α to alleviate disuse skeletal muscle atrophy in mice. Chin. J. Biochem. Mol. Biol. 41 (1), 136–146. 10.13865/j.cnki.cjbmb.2024.12.1407

[B31] He W. T. Sun Q. X. Shi X. L. (2008). Research progress on Tai Chi and primary osteoporosis. Chin. J. Osteoporosis, 14 (8), 595–598. 10.3969/j.issn.1006-7108.2008.08.013

[B11] Kanis J. A. Burlet N. Cooper C. Delmas P. D. Reginster J. Y. Borgstrom F. (2008). European guidance for the diagnosis and management of osteoporosis in postmenopausal women. Osteoporos. Int. 19 (4), 399–428. 10.1007/s00198-008-0560-z 18266020 PMC2613968

[B12] Kistler-Fischbacher M. Weeks B. K. Beck B. R. (2020). The effect of exercise intensity on bone in postmenopausal women (part 1): a systematic review. Bone 143, 115696. 10.1016/j.bone.2020.115696 33357833

[B13] Koevska V. (2017). The role of exercise in women with postmenopausal osteoporosis. Osteoporos. Int. (Suppl. 1), 28.

[B34] Li F. Li W. Li L. Wang Y. (2015). Research progress on exercise prescriptions for sarcopenia and osteoporosis. Chin. J. Rehabil. Theo. Prac. 21 (1), 37–41. 10.3969/j.issn.1006-9771.2015.01.015

[B14] Ma Y. X. Guo Q. Hou A. A. (2015). Research progress on exercise prevention and treatment of osteoporosis in the older adult. Chin. J. Osteoporos. 21 (11), 1403–1407. 10.3969/j.issn.1006-7108.2015.11.020

[B15] Matteo P. Rodrigues I. B. Zeinab H. Moore A. Harris S. Bennell K. (2020). Progressive resistance training for improving health-related outcomes in people at risk of fracture: a systematic review and meta-analysis of randomized controlled trials. Phys. Ther. 101, pzaa221. 10.1093/ptj/pzaa221 33367736

[B16] Mikó I. (2018). Effect of a complex exercise programme on postural balance, endurance and falls in women with established osteoporosis. 10.14232/phd.9925 29767227

[B17] Mohebbi R. Shojaa M. Kohl M. Heshmat R. Larijani B. Shab-Bidar S. (2023). Exercise training and bone mineral density in postmenopausal women: an updated systematic review and meta-analysis of intervention studies with emphasis on potential moderators. Osteoporos. Int. 34, 1145–1178. 10.1007/s00198-023-06682-1 36749350 PMC10282053

[B18] Morin S. N. Feldman S. Funnell L. Giangregorio L. Kim S. McDonald-Blumer H. (2023). Clinical practice guideline for management of osteoporosis and fracture prevention in Canada:2023 update. CMAJ 195 (39), E1333–E1348. 10.1503/cmaj.221647 37816527 PMC10610956

[B19] Nelson M. E. Rejeski M. E. Blair S. N. (2007). Physical activity and public health in older adults: recommendation from the American College of Sports medicine and the American heart association (ACSM/AHA). Geriatr. Nurs. 28 (6), 339–340. 10.1016/j.gerinurse.2007.10.002 17762378

[B20] Salari N. Ghasemi H. Mohammadi L. Behzadi M. H. Rabieenia E. Shohaimi S. (2021). The global prevalence of osteoporosis in the world: a comprehensive systematic review and meta-analysis. J. Orthop. Surg. Res. 16 (1), 609. 10.1186/s13018-021-02772-0 34657598 PMC8522202

[B21] Sangtarash F. Shadmehr A. Choobsaz H. Fereydounnia S. Sadeghi A. Jung F. (2025). Effects of resistance training on microcirculation of bone tissue and bone turnover markers in postmenopausal women with osteopenia or osteoporosis: a systematic review. Clin. Hemorheol. and Microcirc. 89 (2), 171–180. 10.1177/13860291241291411 39973439

[B22] Tian X. Zhao R. Q. Peng Y. (2023). Key principles and applications of exercise prescriptions for osteoporosis prevention. Chin. J. Gerontology 43 (13), 3324–3327. 10.3969/j.issn.1005-9202.2023.13.062

[B23] Ubago-Guisado E. Sánchez-Sánchez J. Vila-Maldonado S. Martínez-González M. A. García-Hermoso A. (2019). Effects of Zumba® and aquagym on bone mass in inactive middle-aged women. Medicina 55 (1), 23. 10.3390/medicina55010023 30669665 PMC6358983

[B24] Wang X. Wang X. W. Zhu T. (2024). Research progress on the effects of high-intensity resistance training on bone mineral density and bone turnover markers in osteoporosis patients. Chin. Community Dr. 40 (36), 5–7. 10.3969/j.issn.1007-614x.2024.36.003

[B25] Wen H. J. Huang T. H. Li T. L. Chong P. N. Ang B. S. (2016). Effects of short-term step aerobics exercise on bone metabolism and functional fitness in postmenopausal women with low bone mass. Osteoporos. Int. 28 (2), 539–547. 10.1007/s00198-016-3759-4 27613719

[B26] Xiao Z. F. Lin D. K. He J. B. (2018). Vertebral osteoporosis and endplate remodeling lead to intervertebral disc degeneration in ovariectomized mice. Chin. J. Osteoporos. 24 (10), 6. 10.3969/j.issn.1006-7108.2018.10.004 PMC6131954 30201052

[B27] Yin X. Y. Wen D. T. Li H. Y. Gao Z. Q. Gao Y. Hao W. (2024). Endurance exercise attenuates Gαq-RNAi induced hereditary obesity and skeletal muscle dysfunction via improving skeletal muscle Srl/MRCC-I pathway in Drosophila. Sci. Rep. 14 (1), 28207. 10.1038/s41598-024-79415-x 39548180 PMC11568267

[B30] Zhang Z. L. Jin X. L. Xia W. B. (2017). Key points interpretation of guidelines for the diagnosis and treatment of primary osteoporosis (2017 Edition). Chin. J. Osteoporosis Bone Mine. Dis. 10.3969/j.issn.1674-2591.2017.05.001

[B28] Zhou Y. Shi C. (2025). Drug use for osteoporosis in middle-aged and older adult people—interpretation of the Canadian clinical practice guidelines for the management of osteoporosis and fracture prevention (2023 edition). Chin. J. Geriatrics 44 (02), 141–148. 10.3760/cma.j.issn.0254-9026.2025.02.006

[B32] Zhu Y. Chen S. Y. Wu Y. P. Zhang J. Li E. J. Li N. (2014). Effect of Tai Chi exercise on lumbar muscle strength in female patients with primary osteoporosis. Chin. J. Osteoporosis. 10.3969/j.issn.1006-7108.2014.01.010

[B29] Zong X. Y. Zong Q. X. Wang X. F. (2023). Summary of the best evidence for exercise intervention in older adults with osteoporosis. J. Nurs. 30 (12), 56–61. 10.16460/j.issn1008-9969.2023.12.056

[B33] Zou J. Zhang L. Ren H. (2015). Expert consensus on exercise for the prevention and treatment of osteoporosis. Chin. J. Osteoporosis, 21 (11), 1305–1311. 10.3969/j.issn.1006-7108.2015.11.001

